# Overlap of Physical, Cognitive, and Social Frailty Affects Ikigai in Community-Dwelling Japanese Older Adults

**DOI:** 10.3390/healthcare10112216

**Published:** 2022-11-04

**Authors:** Soma Tsujishita, Masaki Nagamatsu, Kiyoshi Sanada

**Affiliations:** 1 Department of Physical Therapy, Faculty of Rehabilitation, Kobe International University, 9-1-6 Koyocho-naka, Higashinada-ku, Kobe 658-0032, Hyogo, Japan; 2 Faculty of Research Organization of Science and Technology, Ritsumeikan University, 1-1-1 Noji Higashi, Kusatsu 525-8577, Shiga, Japan; 3 Faculty of College of Sport and Health Science, Ritsumeikan University, 1-1-1 Noji Higashi, Kusatsu 525-8577, Shiga, Japan

**Keywords:** frailty, physical, cognitive, social, ikigai

## Abstract

This study aimed to investigate whether the overlap of physical, cognitive, and social frailty affects Ikigai in community-dwelling Japanese older adults. Participants were 116 community-dwelling older adult Japanese men and women. Associations of physical, cognitive, and social frailty with falls, daily living assessment, and Ikigai were analyzed by group comparisons and multivariate analyses. Physical, cognitive, and social frailty were associated with the risk of falls and Ikigai. An increase in the number of frailty category overlaps was associated with an increased risk of falls and decrease in Ikigai. Multivariate analyses adjusted for confounding factors showed that physical and cognitive frailty were related to Ikigai. In conclusion Two or more overlapping numbers of physical, cognitive, and social frailty had adverse effects on Ikigai in community-dwelling Japanese older adults.

## 1. Introduction

The prevention of frailty contributes to an extension of healthy life expectancy and the reduction of health care costs [1]. The concept of frailty encompasses physical problems and mental and psychological problems such as cognitive dysfunction and depression, as well as social problems such as living alone and economic deprivation [2]. Physical frailty, psychiatric/psychological frailty, and social frailty have each been shown to contribute to an increased risk of falls and impairment of basic activities of daily living (ADL) [1]. In particular, falling has been shown to impact QOL, and leads to negative consequences such as the avoidance of daily living activities and social contact and the promotion of anxiety and depression, causing long-term physical disability [2].

A systematic review of transition rates from physical frailty to healthy physical function among community-dwelling older adults reported that, over a mean follow-up period of 3.9 years, only one-quarter of the older adults who were physical prefrailty at baseline improved to healthy status, a very small number [3]. This suggests that not only frailty but also prefrailty are important factors which impact the improvement of health status of the older adults.

Successful aging is also important for extending healthy life expectancy. Successful aging is a state of low risk of disease and disease-related disability, high physical and mental function, and active involvement in society [4]. The Purpose In Life (PIL) test [5], the Philadelphia Geriatric Center (PGC) Morale Scale [6], and subjective well-being [7] have been used as indicators of successful aging, and recently, the importance of Ikigai (meaning for life) has been recognized [8]. Ikigai differs from other indicators of successful aging. Specifically, while other indicators focus on personal life satisfaction and happiness, Ikigai is a comprehensive concept that includes satisfaction with social relationships in addition to personal satisfaction and happiness [9]. This focus on satisfaction with social relationships is similar to the concept of successful aging mentioned above and is the reason why Ikigai is important. In particular, East Asians, including Japanese, tend to be more intrinsically motivated when their friends and family members choose their goals, while Westerners tend to be more intrinsically motivated when they themselves choose their goals [10], indicating that the achievement of goals is related to successful aging [11]. Therefore, increasing Ikigai, a measure of satisfaction with social relationships, is important.

With regard to the relationship between Ikigai and health status, associations with ADL impairment [12], participation in care prevention and community activities [13], and health-related lifestyle habits (exercise, diet, sleep, and other habits) [14] have been reported among community-dwelling older adults. However, to our knowledge, no study to date has examined the relationship between comprehensive frailty, including physical, cognitive, and social frailty, and Ikigai. If an association between frailty and Ikigai is detected, then it becomes possible to consider interventions for frailty that can enhance Ikigai.

Against this backdrop, the present study aimed to investigate whether the overlap of physical, cognitive, and social frailty affects Ikigai in community-dwelling older adults.

## 2. Materials and Methods

### 2.1. Study Design

This cross-sectional survey of older adults residents in the region was conducted between June 2021 and November 2021.The survey targets were informed orally about the purpose and content of the study, that participation in the study was voluntary, that there would be no disadvantage if they did not fill out the questionnaire, that they could stop the study even after consent to cooperate, and that there would be no disadvantage in that case either. They were informed that the data would be processed statistically, so the respondents would not be identified. The respondents’ consent to cooperate in this survey was obtained by their signing the consent form. This study was approved by the Ritsumeikan University Ethics Review Committee for Medical Research Involving Human Subjects (review number: BKC-LSMH-2021-011) and the Nara Gakuen University Research Ethics Review Committee (receipt number: 2–019).

### 2.2. Participants

The characteristics of the participants, inclusion and exclusion criteria are described below, and shown in a flowchart of the participants. We recruited 116 older adult residents of Usa City (Oita Prefecture) and Kashiwara City (Osaka Prefecture) who consented to participate in this study (23 males and 93 females, mean age ± standard deviation 75.1 ± 5.7 years).

Exclusion criteria were as follows.

1. Persons infected or suspected of being infected with COVID-19.
2. Those who had difficulty completing the questionnaire due to cognitive decline.
3. Those who were certified as requiring nursing care insurance (nursing care insurance 1 or higher).
4. Those who had a history of mental illness.
5. Those who were undergoing orthopedic surgery or had limited mobility.
6. Those who were ill and judged to be ineligible by the study physician to participate.

**Figure 1:**
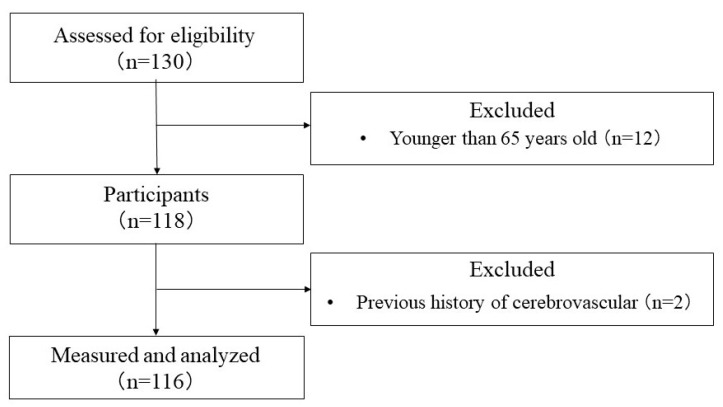
Flowchart of the selection process of the study participants.

Data from 116 participants were ultimately subjected to statistical analysis (as shown in Figure 1).

### 2.3. Sample Size Calculation

Using G*Power 3.1 software (Heinrich Heine University, Düsseldorf, Germany), the sample size for frailty was calculated as power 80%, alpha error 0.05, and effect size 0.40 (large). The number of participants required for this study was found to be 66. To account for the possibility of participant attrition, 130 participants were recruited.

### 2.4. Outcomes

Data on basic information (age, gender, height, weight, BMI, and underlying diseases), physical frailty, cognitive frailty, social frailty, fall risk, ADL, and Ikigai were obtained from participants.

Physical frailty was assessed using J-CHS criteria [15], which include five items: weight loss, fatigue, decreased physical activity, decreased grip strength, and decreased walking speed. If three or more of these items applied, the patient was considered to be physically frail, and if only one or two items apply, the patient was considered to be physically prefrail, i.e., the stage before physical frailty.

Cognitive frailty was defined as a combination of subjective cognitive decline and physical prefrailty, based on previous research [16]. Methods for assessing cognitive frailty, physical frailty and reduced gait speed are used to assess physical function decline. As for cognitive decline, such as mild cognitive impairment (MCI), some studies use objective cognitive decline, some use subjective cognitive decline, and some use a clinical dementia rating (CDR) of 0.5; others have reported variation among studies [17]. The prevalence of cognitive frailty has also been reported to vary by population, ranging from 1.0 to 39.7% [17]. The reason for using the combination of subjective cognitive decline and physical prefrailty as the assessment of cognitive frailty in this study was that using the combination of objective cognitive decline and physical frailty could have resulted in a much lower prevalence rate. In a previous study, the prevalence of cognitive frailty, defined by physical frailty and objective cognitive decline, was 1.2% [18,19]. Another advantage is that the assessment of subjective cognitive function is less burdensome for the subject, who is an older adult person. Subjective cognitive decline was defined as those who answered “Yes” to the Geriatric Depression Scale 15 (GDS15) question “ Do you feel you have more problems with memory than most?” [16].

Social frailty was defined as frailty for two or more of the following five items and prefrailty for one item: “I go out less frequently than last year (yes),” “I visit friends (no),” “I think I am useful to friends and family (no),” “I live alone (yes),” and “I have a conversation with someone every day (no) [20]”. Social frailty was divided into three groups (social frailty, social prefrailty, and robust).

The Locomotion 5 (Locomo5), chair stand test, Timed Up and Go Test (TUG), and the 2-step test were used to assess fall risk. Locomo5 is a 5-item questionnaire rated on a scale from 0 (no difficulty), 1 (slight difficulty), 2 (moderate difficulty), 3 (considerable difficulty), and 4 (severe difficulty), with a maximum score of 20 and higher scores indicating more limitations in mobility [21]. The chair stand test (CST) used a 42-cm pipe chair. Participants were asked to complete five rises from the chair starting from a sitting position, and the time required until the last sitting was completed was measured. The better of two measurements was analyzed as the measured value [22]. TUG measures the time required to stand up from a chair-sitting position, walk normally to a landmark 3 m ahead, change direction at the landmark, walk back with a normal gait, and then re-sit in a chair [23]. The two-step test was performed as follows: toes were aligned at the starting line, two maximum effort steps were taken, the distance to the toes from the starting line with both feet together was measured, and the two-step value was obtained by dividing the distance of the two steps by the height of the participant [24].

The Kihon Checklist was developed by the Japanese Ministry of Health, Labor, and Welfare to assess frailty in older adults and initiate appropriate care needs [25]. There are 25 questions regarding physical strength, nutrition, eating, socialization, memory, mood, and lifestyle answered on a yes/no basis and scored as 0 (pass) or 1 (fail). The maximum score of 25 indicates severe frailty. In the present study, only an ADL assessment was conducted, so the five items pertaining to ADL were used.

The Ikigai scale for the Elderly (the K-1 Scale) was used to assess Ikigai. The K-1 Scale consists of 16 items and four sub-factors: “self-actualization and motivation,” “sense of fulfillment,” “will to live,” and “sense of being.” The questions are answered on a yes (2 points), neither (1 point), or no (0 points) scale, and the total score is used as a score for the sense of purpose in life [26]. The highest score is 32 (16 × 2), with a higher score indicating higher Ikigai.

### 2.5. Data Analysis

All data are presented as mean ± SD. The following confounding factors that may be associated with frailty were assessed: exercise habits (at least 2 days per week, average exercise time of at least 30 min), educational background (6–9 years or 10–13 years), work status, financial comfort, marriage (spouse or bereaved/separated or never married), falls (in past year), hospitalization (in past year), and depression (GDS 15). Values and scores of each assessment item were compared between groups using the χ^2^ test after cross-tabulation, the Kruskal–Wallis test, and the Mann–Whitney U test. A binomial logistic regression analysis using the forced entry method was conducted, with Ikigai (high Ikigai group, low Ikigai group) as the dependent variable and age, gender, physical frailty (pre-frailty or higher), cognitive frailty, and social frailty (pre-frailty or higher) as independent variables. Based on the results of the χ^2^ test, Kruskal–Wallis test, and Mann–Whitney U test, independent variables were extracted from each measurement item, and a forced entry binomial logistic regression analysis was performed with physical frailty (prefrail and above as frail), cognitive frailty, and social frailty (prefrail and above as frail) as dependent variables. SPSS version 27 for Windows (IBM) was used for statistical analyses, with the significance level set at 5%.

## 3. Results

The characteristics of the participants are shown in Table 1. The prevalence of physical frailty, prefrailty, and robust were 13 (11.2%), 60 (51.7%), and 43 (37.1%), respectively. The prevalence of cognitive frailty was 39 (33.6%), and robust was 77 (66.4%). The prevalence of social frailty, prefrailty, and robust were 55 (47.4%), 24 (20.7%), and 37 (31.9%), respectively (shown in Table 1).

In between-group comparisons by each frailty, physical frailty was associated with fall risk assessment (2-step value (*p* = 0.000, *p* < 0.001), locomo5 (*p* = 0.000, *p* < 0.001), chair stand test (*p* = 0.011, *p* < 0.05)), ADL (*p* = 0.042, *p* < 0.05), and Ikigai (*p* = 0.000, *p* < 0.001) (shown in Table 2). Cognitive frailty was associated with fall risk assessment (locomo5 (*p* = 0.000, *p* < 0.001), chair stand test (*p* = 0.003, *p* < 0.05)) and Ikigai (*p* = 0.000, *p* < 0.001) (shown in Table 3). Social frailty was associated with fall risk assessment (2-step value (*p* = 0.007, *p* < 0.05), locomo5 (*p* = 0.000, *p* < 0.001), chair stand test (*p* = 0.000, *p* < 0.001)), ADL (*p* = 0.001, *p* < 0. 001), and Ikigai (*p* = 0.000, *p* < 0.001) (shown in Table 4). Multiple comparisons of variables that showed significant associations indicated that physical frailty, cognitive frailty, and social frailty had a significant negative impact on fall risk and Ikigai compared to being robust.

**Figure 2:**
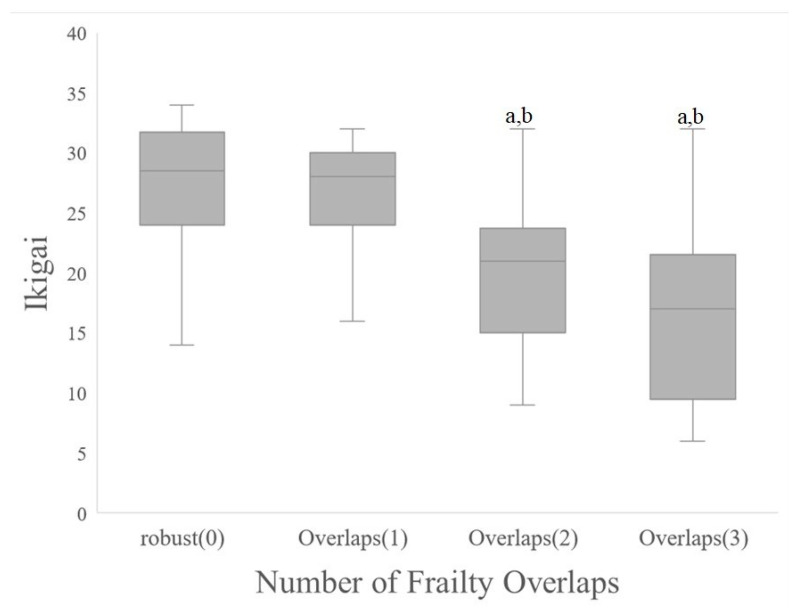
Comparison of life fulfillment by number of frailty overlaps. (a) *p* < 0.001 vs. robust(0); (b) *p* < 0.001 vs. Number of Frailty Overlaps(1).

Between-group comparisons of each item by the number of frailty category overlaps showed associations with fall risk assessment (2-step value (*p* = 0.002, *p* < 0.05), locomo5 (*p* = 0.000, *p* < 0.001), chair stand test (*p* = 0.002, *p* < 0.05)), and Ikigai (*p* = 0.000, *p* < 0.001). Multiple comparisons of variables that showed significant associations indicated that physical frailty, cognitive frailty, and social frailty had a significant negative impact on Ikigai compared to being robust (shown in Figure 2).

We next conducted binomial logistic regression analysis using the forced entry method, with age, gender, physical frailty, cognitive frailty, and social frailty as independent variables, and Ikigai as the dependent variable. Ikigai was significantly associated with physical frailty (odds ratio (OR): 2.67, 95% confidence interval (95% CI): 1.09–6.55), cognitive frailty (OR: 3.26, 95% CI: 1.36–7.82), and social frailty (OR: 2.93, 95% CI: 1.14–7.51) (*p* < 0.05). Hosmer and Lemeshow’s test was significant at *p* > 0.05, and the goodness of fit of the logistic regression model was 75.8%.

Next, binomial logistic regression analysis using the forced entry method was conducted using items showing significant differences between groups as independent variables and physical, cognitive, and social frailty as dependent variables. Physical frailty (*p* < 0.05) was significantly associated with Ikigai (OR: 0.88, 95% CI: 0.80–0.97). Hosmer and Lemeshow’s test was significant at *p* > 0.05, with a target accuracy of 69.8%, indicating a good fit for the logistic regression model. Cognitive frailty was also significantly associated with Ikigai (OR: 0.88, 95% CI: 0.81–0.95). Hosmer and Lemeshow’s test was significant at *p* > 0.05, with a target accuracy of 73.3%, indicating a good fit for the logistic regression mode. Social frailty was also significantly associated with ADL (OR: 1.95, 95% CI: 1.004–3.79). Hosmer and Lemeshow’s test was significant at *p* > 0.05, with a target accuracy of 77.6%, indicating a good fit for the logistic regression model.

## 4. Discussion

The present study examined whether the overlap between physical, cognitive, and social frailty affects Ikigai in community-dwelling older adults. In summary, the results of this study suggest that physical frailty, cognitive frailty, and social frailty are associated with fall risk and Ikigai and that the overlap of two or more frailty categories has a negative impact on fall risk and Ikigai. Multivariate analysis suggested that physical frailty and cognitive frailty were associated with Ikigai.

In previous studies on the prevalence of physical, cognitive, and social frailty among community-dwelling older adults in Japan, the prevalence of physical frailty was 11% for frailty and 57% for prefrailty [15]. The prevalence of cognitive frailty has also been reported to vary by population, ranging from 1.0 to 39.7% [17], and the prevalence of social frailty was 11% for frailty and 25% for prefrailty [20]. In the present study, the prevalence of physical frailty was 11% for frailty and 52% for prefrailty, cognitive frailty was 34%, and social frailty was 47% for frailty and 21% for prefrailty, indicating that the prevalence of physical frailty and cognitive frailty was similar to previous studies, but that of social frailty somewhat differed. For social frailty, our study was conducted from July to November 2021, which coincides with the COVID-19 pandemic. Accordingly, the prevalence of social frailty may have increased due to the impact of the pandemic, which led to restrictions on outings.

In comparisons by frailty category, physical, cognitive, and social frailty were associated with the number of underlying diseases, risk of falls, depression, and Ikigai. Physical frailty was associated with exercise habits, while cognitive frailty was associated with falls. Social frailty was associated with older age, ADL impairment, exercise habits, living alone, and having no spouse. Previous studies have reported associations between each of these frailty categories and each of the factors, with similar results [1,15,20,27,28].

Although many of our results were similar to those of previous studies, one unique finding was that physical, cognitive, and social frailty were each associated with Ikigai, indicating that each frailty category also affects a higher mental function. In other countries, quality of life (QOL) is a concept similar to Ikigai. The World Health Organization (WHO) defines QOL as “an individual’s perception of his or her life situation about goals, expectations, standards or interests within the culture and values in which he or she lives [29].” Ikigai is considered a major factor related to QOL in the older adults [30], and it can be inferred that fulfillment of life and QOL are related. With regard to the association between QOL and frailty, a previous study conducted in the Netherlands reported that QOL was associated with physical, psychological, and social frailty [31]. Another concept similar to Ikigai is “purpose in life”. The older adults with a high sense of purpose tend to have goals and aspirations for the future and find meaning in their daily activities, according to reports [32]. It has also been suggested that people with a greater sense of purpose are more likely to engage in activities such as exercise and social participation, which may prevent dementia risk [33]. In other words, in this study, those who have Ikigai have goals for the future and find meaning in their daily activities, which are more likely to induce activities such as exercise and social participation, and these factors may account for the low percentage of those with physical, cognitive and social frailty.

Next, in the group comparison by the number of overlaps of each type of frailty, we found that two or more overlapping frailty counts were associated with age, number of underlying diseases, risk of falls, depression, and Ikigai. To our knowledge, no previous study has examined the association between the number of frailty category overlaps and adverse events. A longitudinal study of 2406 community-dwelling older adults followed for 3 years reported that a combination of physical and social frailty measures could more accurately identify individuals at increased risk of functional impairment than physical or social frailty alone [28]. There are also several reports on the interrelationship between each frailty category, with social frailty directly affecting the increase in physical frailty [34]. In addition, studies have shown that the number of overlaps between physical, cognitive, and social frailty did not affect ADL disability, QOL, or risk of hospitalization [35]. Although overlaps between physical, cognitive, and social frailty have been shown to negatively impact adverse events, there have been no reports comparing these frailty categories based on the number of overlaps, as was done in the present study. Studies which evaluate the number of overlaps in the three frailty categories in the field of long-term care prevention with the purpose of predicting the prognosis for adverse events are warranted.

Multivariate analyses conducted with Ikigai as the dependent variable and age, gender, and each of the frailty variables as independent variables showed significant differences in Ikigai between physical, cognitive, and social frailty. In order to clarify factors related to each frailty category, we conducted a multivariate analysis in which variables that showed significant differences between groups were included. The results of this analysis suggest that physical and cognitive frailty is related to Ikigai. Although previous studies have suggested a relationship between physical and cognitive functions and Ikigai [12], we could not identify studies that directly investigated frailty and the sense of purpose in life. Physical and cognitive frailty interventions will likely be important to improve Ikigai and extend healthy life expectancy.

Limitations and issues of this study include the fact that the study was conducted from July to November 2021, and the COVID-19 pandemic was expected to have a negative impact on frailty, risk of falling, ADL impairment, and Ikigai, resulting in a low overall figure. Furthermore, since the sample size was small in this study, future studies will need to increase the sample size and consider which combinations of each variable affect adverse events. In addition, cognitive frailty was defined as a combination of subjective cognitive decline and physical prefrailty, based on previous studies. This was due to the predicted lower prevalence when assessed by objective cognitive decline and also because of the burden on the study subjects. However, we believe that future studies will be able to validate their findings more reliably by assessing objective cognitive decline. Finally, there is no access to the causal relationship between the different subtypes of frailty and Ikigai.

## 5. Conclusions

We found that two or more overlapping numbers of physical, cognitive, and social frailty had adverse effects on Ikigai in community-dwelling Japanese older adults. These findings may contribute to efforts to prevent frailty and maintaining and improving the well-being of this population.

## Figures and Tables

**Table 1:** Characteristics of the participants.

Age (years)	75.1 ± 5.7	
BMI (kg/m^2^)	23.1 ± 3.1	
2-step value (2 strides/height)	1.1 ± 0.2	
Locomo5 (points)	3.6 ± 3.6	
Chair stand test (seconds)	10.1 ± 3.8	
TUG (seconds)	6.8 ± 1.3	
GDS (points)	3.6 ± 3.1	
Ikigai (points)	22.1 ± 7.3	
ADL (points)	0.7 ± 1.1	
Number of underlying diseases (persons)	None: 37, One: 58, Two: 17, Three: 2, Four: 2	
Gender (persons)	Male: 23, Female: 93	
Educational background (persons)	6–9 years: 8, 10–13 years: 108	
Financial comfort (persons)	Yes: 40, No: 76	
Family (persons)	Living alone: 23, living together: 93	
Work (persons)	Have done: 23, Have not done: 93	
Married (persons)	Spouse: 74, Bereaved/separated: 38, Never married: 4	
Exercise habits (persons)	Yes: 77, No: 39	
Falls (persons)	Yes: 33, No: 83	
Hospitalization (persons)	Yes: 22, No: 94	
Physical Frailty (persons)	Robust: 43, Pre-frailty: 60, Frailty: 13	
Cognitive Frailty (persons)	Robust: 77, Frailty: 39	
Social frailty (persons)	Robust: 37, Pre-frail: 24, Frailty: 55	

Mean ± standard deviation, BMI: Body Mass Index, TUG: Timed Up & Go test. GDS: Geriatric Depression Scale, ADL: Activities of Daily Living.

**Table 2:** Comparison of groups classified by physical frailty.

	Physical Frailty ^(1)^					
Evaluation Item	a. Robust (n = 43)	b. Pre-Frailty (n = 60)	c. Frailty (n = 13)	*p*-Value	*p* ^(2)^	Multiple Comparisons
Age	73.0 (69.0–78.5)	76.5 (72.0–80.0)	77.0 (76.0–79.0)	0.052		
BMI	23.5 (21.8–25.0)	22.5 (21.1–24.2)	22.8 (20.4–23.9)	0.412		
GDS	2.0 (0.0–4.0)	3.0 (2.0–7.0)	5.0 (3.0–9.0)	0.000	***	a < c, a < b
Number of underlying diseases	1.0 (0.0–1.0)	1.0 (0.0–1.0)	1.0 (1.0–2.0)	0.018	*	a < c
2-step value	1.2 (1.1–1.3)	1.1 (1.0–1.2)	1.0 (0.9–1.1)	0.000	***	a > c, a > b
Loco5	1.0 (0.0–3.0)	3.5 (1.0–6.5)	5.0 (3.0–6.0)	0.000	***	a < b, a < c
5-stance test	8.7 (7.7–9.6)	9.5 (8.0–12.5)	9.5 (9.0–11.8)	0.011	*	a < b, a < c
TUG	6.5 (5.9–7.3)	6.6 (6.0–7.6)	6.8 (6.5–8.2)	0.284		
ADL	0.0 (0.0–1.0)	0.0 (0.0–1.0)	1.0 (0.0–2.0)	0.042	*	a < c
Self-actualization and motivation	9.0 (7.5–12.0)	8.0 (5.0–10.0)	7.0 (2.0–8.0)	0.002	*	a > b, a > c
Life fulfillment	9.0 (7.5–10.0)	7.0 (4.0–8.5)	7.0 (4.0–8.0)	0.000	***	a > b, a > c
Willingness to live	4.0 (3.0–4.0)	3.0 (2.0–4.0)	3.0 (2.0–4.0)	0.008	*	a > b
Sense of presence	6.0 (5.0–6.0)	4.0 (3.0–6.0)	3.0 (0.0–5.0)	0.000	***	a > b, a > c
Ikigai	28.0 (23.0–31.0)	19.0 (15.0–26.0)	19.0 (10.0–23.0)	0.000	***	a > b, a > c
Male n, %	8, 18.6	11, 18.3	4, 30.8	0.578		
Have an exercise habit n, %	34, 79.1	37, 61.7	6, 46.2	0.031	*	
Have 10–13 years of education n, %	42, 97.7	54, 90.0	12, 92.3	0.566		
Have a job n, %	11, 25.6	12, 20.0	0, 0.0	0.064		
Financially well-off n, %	17, 39.5	17, 28.3	6, 46.2	0.358		
Living alone n, %	5, 11.6	15, 25.0	3, 23.1	0.245		
Married n, %	33, 76.7	34, 56.7	7, 53.8	0.349		
Have had a fall n, %	8, 18.6	18, 30.0	7, 53.8	0.034	*	
Have been hospitalized n, %	6, 14.0	14, 23.3	2, 15.4	0.406		

Median (interquartile range), BMI: Body Mass Index, TUG: Timed Up & Go test, GDS: Geriatric Depression Scale, ADL: Activities of Daily Living. ^(1)^ Physical frailty was compared among the three groups by the Kruskal–Wallis test, and subsequent multiple comparisons were made using the Mann–Whitney U test, with Bonferroni’s correction (*p* < 0.05/3 = 0.017) to account for multiplicity (with significant differences between different alphabets). For the nominal scale, the χ^2^ test and Fisher’s direct method were used. ^(2)^ * *p* < 0.05, *** *p* < 0.001.

**Table 3:** Comparison of groups classified by cognitive frailty.

		Cognitive Frailty ^(1)^			
	Evaluation Item	Robust (n = 77)	Frailty (n = 39)	*p*-Value	*p* ^(2)^
	Age	74.0 (70.0–78.0)	76.0 (71.5–80.0)	0.101	
	BMI	22.8 (21.4–25.1)	23.3 (21.8–24.2)	0.568	
	GDS	1.0 (0.0–3.0)	6.0 (3.0–7.0)	0.000	***
	Number of underlying diseases	1.0 (0.0–1.0)	1.0 (1.0–1.5)	0.016	*
Falls Index	2-step value	1.1 (1.1–1.3)	1.1 (1.0–1.2)	0.105	
	Loco5	1.0 (0.0–4.0)	4.0 (2.0–6.5)	0.000	***
	5-stance test	8.8 (7.7–9.8)	10.1 (8.5–12.5)	0.003	*
	TUG	6.5 (6.0–7.6)	6.6 (5.9–7.6)	0.685	
	ADL	0.0 (0.0–1.0)	0.0 (0.0–1.0)	0.544	
Ikigai Index	Self-actualization and motivation	9.0 (7.0–11.0)	7.0 (4.0–10.0)	0.002	*
	Life fulfillment	9.0 (7.0–10.0)	6.0 (4.0–8.0)	0.000	***
	Willingness to live	4.0 (3.0–4.0)	3.0 (2.0–4.0)	0.000	***
	Sense of presence	5.0 (4.0–6.0)	4.0 (2.0–5.0)	0.000	***
	Ikigai	26.0 (20.0–30.0)	19.0 (13.0–24.0)	0.000	***
	Male n, %	17, 22.1	6, 15.4	0.105	
	Have an exercise habit n, %	56, 72.7	21, 53.8	0.431	
	Have 10–13 years of education n, %	74, 96.1	34, 87.2	0.167	
	Have a job n, %	18, 23.4	5, 12.8	0.076	
	Financially well-off n, %	25, 32.5	15, 38.5	0.421	
	Living alone n, %	13, 16.9	10, 25.6	0.172	
	Married n, %	55, 71.4	19, 48.7	0.366	
	Have had a fall n, %	16, 20.8	17, 43.6	0.046	*
	Have been hospitalized n, %	15, 19.5	7, 17.9	0.342	

Median (interquartile range), BMI: Body Mass Index, TUG: Timed Up & Go test. GDS: Geriatric Depression Scale, ADL: Activities of Daily Living. ^(1)^ Mann–Whitney U test was performed for cognitive frailty. For nominal measures, the χ^2^ test and Fisher’s direct method were used. ^(2)^ * *p* < 0.05, *** *p* < 0.001.

**Table 4:** Comparison of groups classified by social frailty.

		Social Frailty ^(1)^					
	Evaluation Item	a. Robust (n = 37)	b. Pre-Frailty (n = 24)	c. Frailty (n = 55)	*p*-Value	*p* ^(2)^	Multiple Comparisons
	Age	74.0 (70.0–78.0)	72.0 (69.5–77.0)	78.0 (73.0–80.0)	0.003	*	a < c, b < c
	BMI	23.6 (22.0–25.1)	23.7 (21.8–24.3)	22.4 (20.7–23.9)	0.247		
	GDS	1.5 (0.0–3.0)	2.0 (0.5–4.5)	4.0 (2.0–7.5)	0.000	***	a < c, b < c
	Number of underlying diseases	1.0 (0.0–1.0)	1.0 (0.0–1.0)	1.0 (1.0–2.0)	0.004	*	a < c, b < c
Falls Index	2-step value	1.2 (1.1–1.3)	1.1 (1.1–1.2)	1.1 (0.9–1.2)	0.007	*	a > c
	Loco5	1.0 (0.0–2.0)	2.0 (0.0–4.0)	5.0 (2.0–8.0)	0.000	***	a < c, b < c
	5-stance test	8.6 (7.6–9.6)	8.9 (7.9–9.5)	10.7 (8.3–13.3)	0.000	***	a < c, b < c
	TUG	6.5 (6.0–6.8)	6.6 (5.7–8.1)	6.6 (6.1–7.6)	0.377		
	ADL	0.0 (0.0–0.0)	0.0 (0.0–1.0)	1.0 (0.0–2.0)	0.001	*	a < c
Ikigai Index	Self-actualization and motivation	9.0 (7.0–11.0)	10.0 (7.0–12.0)	7.0 (4.0–9.5)	0.001	*	a > c, b > c
	Life fulfillment	9.0 (7.0–10.0)	8.5 (7.0–10.0)	6.0 (4.0–8.0)	0.000	***	a > c, b > c
	Willingness to live	4.0 (3.0–4.0)	4.0 (3.0–4.0)	2.0 (2.0–4.0)	0.000	*	a > c, b > c
	Sense of presence	6.0 (5.0–6.0)	6.0 (4.5–6.0)	3.0 (2.0–5.0)	0.000	***	a > c, b > c
	Ikigai	28.0 (23.0–30.0)	26.0 (22.5–30.5)	18.0 (13.0–23.0)	0.000	***	a > c, b > c
	Male n, %	9, 24.3	1, 4.2	13, 23.6	0.178		
	Have an exercise habit n, %	31, 83.8	13, 54.2	33, 60.0	0.029	*	
	Have 10–13 years of education n, %	35, 94.6	24, 100.0	49, 89.1	0.416		
	Have a job n, %	12, 32.4	2, 8.3	9, 16.4	0.035	*	
	Financially well-off n, %	17, 45.9	10, 41.7	13, 23.6	0.095		
	Living alone n, %	2, 5.4	0, 0.0	21, 38.2	0.000	***	
	Married n, %	28, 75.7	20, 83.3	26, 47.3	0.006	*	
	Have had a fall n, %	8, 21.6	6, 25.0	19, 34.5	0.344		
	Have been hospitalized n, %	8, 21.6	4, 16.7	10, 18.2	0.814		

Median (interquartile range), BMI: Body Mass Index, TUG: Timed Up & Go test, GDS: Geriatric Depression Scale, ADL: Activities of Daily Living. ^(1)^ Social frailty was compared among the three groups by the Kruskal–Wallis test, and subsequent multiple comparisons were made using the Mann-–Whitney U test, with Bonferroni’s correction (*p* < 0.05/3 = 0.017) to account for multiplicity (with significant differences between different alphabets). For the nominal scale, the χ^2^ test and Fisher’s direct method were used. ^(2)^ * *p* < 0.05, *** *p* < 0.001.

## Data Availability

Not applicable to this article.
